# The *In Silico* Identification of Potential Members of the Ded1/DDX3 Subfamily of DEAD-Box RNA Helicases from the Protozoan Parasite *Leishmania infantum* and Their Analyses in Yeast

**DOI:** 10.3390/genes12020212

**Published:** 2021-02-01

**Authors:** Molka Mokdadi, Yosser Zina Abdelkrim, Josette Banroques, Emmeline Huvelle, Rafeh Oualha, Hilal Yeter-Alat, Ikram Guizani, Mourad Barhoumi, N. Kyle Tanner

**Affiliations:** 1 Expression Génétique Microbienne, UMR8261 CNRS, Université de Paris, Institut de Biologie Physico-Chimique, 13 rue Pierre et Marie Curie, 75005 Paris, France; molka.mokdadi@gmail.com (M.M.); yosr_abdelkrim@yahoo.fr (Y.Z.A.); josette.banroques@ibpc.fr (J.B.); emmeline.huvelle@ibpc.fr (E.H.); yeter.hilal@yahoo.fr (H.Y.-A.); 2 PSL Research University, 75005 Paris, France; 3 Laboratory of Molecular Epidemiology and Experimental Pathology (LR16IPT04), Institut Pasteur de Tunis, Université de Tunis El Manar, 13 Place Pasteur, BP74 Tunis-Belvédère 1002, Tunisia; oualha_rafeh@yahoo.fr (R.O.); ikram.Guizani@pasteur.tn (I.G.); 4 Institut National des Sciences Appliquées et Technologies, Université de Carthage, CEDEX, Tunis 1080, Tunisia

**Keywords:** *Leishmania*, *Trypanosoma brucei*, Ded1/DDX3, RNA helicase, DEAD-box, leishmaniasis, trypanosomatid, *Saccharomyces cerevisiae*

## Abstract

DEAD-box RNA helicases are ubiquitous proteins found in all kingdoms of life and that are associated with all processes involving RNA. Their central roles in biology make these proteins potential targets for therapeutic or prophylactic drugs. The Ded1/DDX3 subfamily of DEAD-box proteins is of particular interest because of their important role(s) in translation. In this paper, we identified and aligned the protein sequences of 28 different DEAD-box proteins from the kinetoplast-protozoan parasite *Leishmania infantum*, which is the cause of the visceral form of leishmaniasis that is often lethal if left untreated, and compared them with the consensus sequence derived from DEAD-box proteins in general, and from the Ded1/DDX3 subfamily in particular, from a wide variety of other organisms. We identified three potential homologs of the Ded1/DDX3 subfamily and the equivalent proteins from the related protozoan parasite *Trypanosoma brucei*, which is the causative agent of sleeping sickness. We subsequently tested these proteins for their ability to complement a yeast strain deleted for the essential *DED1* gene. We found that the DEAD-box proteins from Trypanosomatids are highly divergent from other eukaryotes, and consequently they are suitable targets for protein-specific drugs.

## 1. Introduction

Leishmaniases are parasitic diseases caused by the protozoan parasite of the genus *Leishmania*. There are currently 54 recognized *Leishmania* species of which at least 21 are human pathogens that are transmitted by the numerous species of female sandflies belonging to the subfamily Phlebotominae [1,2,3]. These parasites have a complex, dimorphic, life cycle that involves a number of metabolic transitions and differentiated forms. The sandflies inject the flagellated *Leishmania* metacyclic promastigotes into the bloodstream while feeding on their hosts. The promastigotes are eventually engulfed by macrophages (and other cell types) through phagocytosis whereupon the parasites transform into immobile amastigotes in the acidic environment of the modified phagolysosome (the parasitophorous vacuole; reviewed by [4]) where they feed and reproduce. The replicating amastigotes can infect other macrophages and other cells when the macrophages are lysed or by other poorly defined mechanisms. Infected macrophages are eventually taken back up by the sandfly during the blood meal whereupon the amastigotes differentiate into extracellular, flagellated, procyclic promastigotes in the midgut of the sandflies. They subsequently divide into metacyclic promastigotes that migrate to the pharyngeal valve of the sandfly for the next round of infection [4,5,6].

*Leishmania* are endemic in more than 98 countries creating serious health problems in many countries around the world. They are manifested in several clinical forms ranging from self-healing skin lesions of cutaneous leishmaniasis (CL) to the more severe visceral leishmaniasis (VL) [7,8,9]. Around 700,000 to 1 million new cases and some 26,000 to 65,000 deaths occur annually (WHO, 2020, [10]). Despite tremendous efforts, no effective vaccine has yet been developed [11,12,13]. Mainstay treatment regimes for leishmaniases are based on chemotherapy involving the use of the pentavalent antimonials. Commonly used second-line drugs are miltefosine, amphotericin B, liposomal amphotericin B and paromomycin. All these treatments require long treatment courses, are toxic and costly, and have adverse effects; moreover, there is the risk of the parasites developing drug resistance [14,15,16]. Thus, there is need to identify new specific targets in order to develop new drugs to control the disease. Due to the availability of the genomic sequence of most *Leishmania* species during the last few years, there are extensive efforts to identify new genes involved in the pathogenicity or that are essential for survival of the parasites that could be the targets for therapeutic or prophylactic compounds.

The DEAD-box family of RNA helicases are ubiquitous proteins found in all kingdoms of life that are involved in all processes involving RNA from transcription to decay (reviewed by [17,18,19]). They are part of the DExD/H-box superfamily 2 (SF2) of putative DNA and RNA helicases that are named after the amino acid sequence of the Walker B motif (motif II) that is involved in NTP binding and hydrolysis. They contain catalytic cores consisting of two, linked, RecA-like domains that have the conserved motifs associated with ligand binding and enzymatic activity, and they have highly variable amino- and carboxyl-terminal sequences and insertions within the catalytic core (reviewed by [20,21]). The DEAD-box proteins are ATP-dependent RNA binding proteins and RNA-dependent ATPases that are capable of unwinding short duplexes in a nonprocessive fashion *in vitro*, but typically at very high protein to substrate ratios. In addition, they can remodel RNA and ribonucleoprotein (RNP) complexes, but they generally have little or no substrate specificity. In contrast, *in vivo* the proteins show a high degree of specificity; for example, the yeast *Saccharomyces cerevisiae* encodes 25 different DEAD-box proteins that are mostly essential and that are not interchangeable—even when overexpressed—with the exception of a single paralog resulting from gene duplication [22,23]. Humans have 37 different proteins [21]. Thus, DEAD-box proteins, and DExD/H-box proteins in general, provide a rich source of potential drug targets (reviewed by [24,25,26,27,28,29]).

The Ded1/DDX3 subfamily of DEAD-box proteins is of particular interest (reviewed by [30,31,32,33]). They are considered translation-initiation factors that are important for 43S ribosome scanning to the initiation complex, but they also shuttle between the nucleus and cytoplasm using the XpoI/CrmI and Mex67/TAP nuclear pore complexes, and they are found in cytoplasmic foci (P-bodies or stress granules) containing translation-inactive mRNAs under stress conditions [34,35,36,37,38]. The yeast *DED1* is an essential gene that is rescued by the plasmid-borne expression of its orthologs from other eukaryotes, including mammalian *DDX3X*, which demonstrates a high degree of functional conservation ([34] and references therein). Due to the important roles these proteins play in the cell, the Ded1/DDX3 subfamily of proteins are considered important targets for therapeutic or prophylactic drugs (reviewed by [25,26,39,40,41,42]). However, it is not clear whether the Ded1/DDX3 subfamily of proteins is actually functionally conserved throughout all the eukaryotes. Moreover, it is not clear what distinguishes the Ded1/DDX3 subfamily from closely related subfamilies such as Vasa/DDX4, which is involved in developmental regulation (reviewed by [43,44,45,46]).

The Kinetoplastida trypanosomatids belong to the Euglenozoa phylum that separated very early from other eukaryotes during evolution [47,48]. Therefore, their genes are expected to be highly divergent from other organisms. Moreover, *Trypanosoma* and *Leishmania* are obligate parasites, which means their genes are subjected to additional divergent forces. Thus, we expect that their encoded proteins will have unique features that will make them particularly useful as targets for the development of protein-specific drugs. In the same vein, we expect comparisons with the homologs from other organisms will reveal highly conserved features that characterize the functionality of the proteins and that will facilitate their identification in other organisms. Moreover, the majority of the *Leishmania* and *Trypanosoma* genes are constitutively expressed as polycistronic RNAs that are subsequently processed into separate capped and polyadenylated mRNAs; thus, translation, and probably DDX3-like proteins, plays a particularly important role in the control of gene expression, especially for the differentiation into the different parasitic forms (reviewed by [49,50,51]). Indeed, we previously showed that the eIF4A-like translation protein, LieIF4A (LINF_010012800/LINF_010012900), is a viable target for therapeutic drugs [52,53].

In this work, we aligned the RecA-like core sequences of 28 of the 29 identified DEAD-box proteins from *L. infantum* and compared them to the consensus sequences of DEAD-box proteins, in general, and to the consensus sequence of the Ded1/DDX3 subfamily in particular. We further analyzed the amino- and carboxyl-terminal sequences for motifs that are characteristic of the Ded1/DDX3 proteins. We then compared the identified candidate proteins to their homologs from *T. brucei*. Finally, we tested the identified proteins from *Leishmania* and *Trypanosoma* for their capacity to complement a yeast strain deleted for the endogenous Ded1 protein at different expression levels and as chimeras of the Ded1 protein. Our results reveal not only the conservation of certain features in these proteins but also the high degree of divergence from other eukaryotes.

## 2. Materials and Methods

### 2.1. Data Acquisition and Analyses

Sequences for *L. infantum* JPCM5 were obtained from the TriTrypDB website (Release 46; https://tritrypdb.org/tritrypdb/) using DEAD-box and helicase as a search terms [54]. These sequences were then compared with those obtained with a Blast of the TriTrypDB website using Ded1 as bait (*E*-value ≤ 4 × 10^−4^). The sequences were subsequently screened for the presence of the conserved motifs that are characteristic of DEAD-box family proteins and that distinguished them from related helicase families. We obtained 29 different genes. The derived protein sequences were then aligned along with representative Ded1/DDX3 proteins on the EMBL-EBI T-coffee server (https://www.ebi.ac.uk/Tools/msa/tcoffee/) using ClustalW with the default setting for the initial alignments [55]. These alignments were subsequently refined by eye to maximize the sequence homology and conserved distances of the motifs using our previous characterizations of DEAD-box proteins as a basis [56]. Phylogenetic trees were derived from the EMBL-EBI T-coffee server using ClustalW and the default settings.

To identify Ded1/DDX3-specific features, we did a Blast search (PMID: 9254694) of the UniProtKB web site (https://www.uniprot.org/) of the Swiss-Prot (release 54.6) and TrEMBL (release 37.6) entries using yeast Ded1 (P06634) as bait [57]. We obtained 250 sequences from various organisms (*E*-value ≤ 4 × 10^−78^), most of which were classified as Ded1/DDX3 or Vasa/DDX4 in the annotations, that were refined to eliminate duplicates, fragments, and proteins that did not conform to the DEAD-box motifs to yield 229 unique sequences. These sequences were aligned as described above. We used the Bock coot-alignment-consensus Perlscript to derive the consensus sequence, which is unfortunately no longer available on the EMBL-Heidelberg server. The consensus sequence yielded most but not all the known motifs of the Ded1/DDX3 subfamily; for example, the eIF4E binding motif was not recovered. Hence, we further refined the alignments using Blast sequences with *E*-values equal to or less than 10^−113^ to yield 96 unique sequences. These sequences were used to obtain the final consensus sequence, but they do not represent the full diversity of sequences of the Ded1/DDX3 subfamily.

The equivalent *T. brucei* 427 and 927 genes were identified by Blasts against the trypanosome genome using the selected *LINF* genes as bait. The proteins identified were equivalent to those listed on the TriTrypDB website as homologs of the corresponding *LINF* genes. The Tb427.05.3600 protein (the long form of Tb927.5.3600) as listed on TriTrypDB had three unidentified amino acids; we sequenced the gene and identified the amino acids as X107V, X280V, and X529C. These sequences were aligned as described above.

The selected proteins were analyzed with PredictProtein (https://www.predictprotein.org) and Pfam (https://pfam.xfam.org/), and they were compared with the results for Ded1 and DDX3 [58,59]. The presence of leucine-rich nuclear export signals (NES) was analyzed with the NetNES 1.1 Server (http://www.cbs.dtu.dk/services/NetNES/) [60]. We further modeled the selected proteins with Swiss-Model using the default setting; the resulting different models were visualized with Swiss-PdbViewer 4.1.0 [61,62,63].

### 2.2. Preparation of L. infantum and T. brucei DNA

We used *L. infantum* LV50 strain (MHOM/TN/94/LV50) that was isolated from a patient suffering from visceral leishmaniasis [64]. The promastigote form was cultured in RPMI-1640/GlutaMAX medium (Gibco BRL, Darmstadt, Germany) complemented with 100 U/mL penicillin, 100 µg/mL streptomycin, and 10% heat-inactivated fetal bovine serum (FBS; Gibco BRL) at 22 °C as previously described [64]. The production of flagellated promastigotes was monitored with a microscope. Parasites at the stationary phase were collected by centrifugation at 4 °C at 3500 rpm (2380 g) in a JOUAN CR3.12 swinging bucket centrifuge for 15 min and stored at −80 °C until needed.

The total genomic DNA was extracted from stationary phase cells. The cell pellet was resuspended in a lysis buffer containing 50 mM NaCl, 10 mM EDTA, 50 mM Tris–HCl, pH 7.5, 100 μg/mL Proteinase K, and 0.5% SDS, and then it was incubated overnight at 55 °C to lyse the cells. The DNA was extracted with phenol and chloroform:isoamyl alcohol (24:1), and then it was precipitated with ethanol as previously described [65]. The concentration of DNA was quantified using the Qubit 2.0 Fluorometer (Invitrogen, Singapore City, Singapore). The *T. brucei* 427 strain DNA was a kind gift from Lucy Glover (Pasteur Institute, Paris, France).

### 2.3. Cloning and Yeast Manipulations

All manipulations of yeast including media preparation, growth conditions, transformation, and 5-fluoroorotic acid (5-FOA) selection, were done according to standard procedures [66]. We used the W303 and BY4742 wildtype yeast strains for all the protein expression experiments. The *∆ded1* yeast strain (*ded1::HIS3*) containing the YCplac33-*DED1* plasmid used in the complementation assays was as previously described [67].

The oligonucleotides used in the cloning are shown in Supplementary Materials Table S1. The selected *LINF* genes were PCR amplified using the indicated oligonucleotides with the purified *L. infantum* or *T. brucei* DNA. PCR reactions were done in a Bio-Rad T100 Thermal Cycler with Q5 High-Fidelity DNA polymerase (New England Biolabs, Évry-Courcouronnes, France) according to the manufacturer’s recommendations. In brief, the amplifications were performed with 1 µL of DNA (20 ng/µL) in a 50 µL volume containing 0.02 U/µL Q5 High-Fidelity DNA polymerase, 1 × buffer, 200 µM dNTPs, 1 × GC enhancer, and 2 µM from each oligonucleotide for 30 cycles. The amplified DNA was digested with SpeI and XhoI for most of the constructs, with SpeI and SalI for *LINF08*, XbaI and SalI for *TRYP08*, and with SpeI and SmaI for *TRYP35*. The digested fragments were purified on a 0.8% agarose gel, eluted with a NucleoSpin Gel and PCR Clean-Up kit (Macherey-Nagel, Düren, Germany) according to the manufacturer’s instructions and cloned into the equivalent sites of the Bluescript plasmid (Stratagene, La Jolla, CA, USA). Final constructs were verified by sequencing and are shown in Supplementary Materials Table S2.

The constructs were subsequently subcloned into the *ADH-2HA*_p415, *ADH-2HA*_p424, and *GPD-2HA*_p424 yeast expression plasmids, and transformed into either the wildtype or *ded1::HIS* yeast strains. Cells were grown in synthetic defined (SD) medium lacking leucine (for p415) or tryptophan (for p424). Cultures of the *ded1::HIS* yeast strain were subsequently serially diluted by a factor of 10 and spotted on SD plates containing 5-FOA and grown at 18 °C, 30 °C, and 36 °C. Proteins from cultures of the wildtype strains were extracted for Western blot analyses.

### 2.4. Synthetic Gene Construction

The synthetic sequence constructs were made by first optimizing the codons to yeast *DED1* using the COOL software that was previously available on the cool.syncti.org server [68]. The proposed sequences were then visually modified to eliminate long strings of homogeneous nucleotides and conflicting restriction sites. The genes were synthesized by Eurofin Scientific (Luxembourg) with 5’ SpeI-NdeI and 3’ XhoI restriction sites. The DNAs were digested with SpeI and XhoI and cloned into the equivalent sites of the *2HA*-p415 and *2HA*-p424 plasmids. Final constructs were all verified by sequencing. The *LINF08Lsyn* and *LINF32syn* had the correct protein sequence. The *LINF35syn* construct introduced an E408K mutation, which was in the amino-terminal sequence outside the catalytic core. The *LINF08Ssyn* was constructed by PCR amplification of the *LINF08Lsyn* with LinJ08_up3 and LinJ08_low2 oligonucleotides containing SpeI, NdeI, and XhoI sites (Supplementary Materials Table S1). The PCR product was digested with SpeI and XhoI, and then cloned into the equivalent sites of the above plasmids. The final constructs were all verified by sequencing.

### 2.5. Ded1 Chimeras of the LINF and TRYP Proteins

The protein chimeras contained the LINF or TRYP RecA-like cores as previously defined [22], using the optimized *LINFsyn* and wildtype *T. brucei* genes, and the amino- and carboxyl-terminal sequences of Ded1. The design took advantage of a conserved aspartic acid (or asparagine in the case of DDX3X) that occurred 7–8 residues upstream of the isolated conserved aromatic group (generally an F) of the Q motif, and a conserved aliphatic group 29–31 residues downstream of motif VI. The final constructs contained the entire RecA-like cores of the LINF and TRYP proteins and the corresponding flanking sequences of Ded1 (1–136, 525–604). Constructs were made with the Gibson Assembly Cloning kit (New England Biolabs) according to the manufacturer’s instructions. Oligonucleotides were designed using the NEBuilder Assembly Tool provided on the New England Biolabs web site and are shown in Supplementary Materials Table S1. As a positive control, we also made a Ded1-DDX3X chimera. All constructs were verified by sequencing.

### 2.6. Western Blot Analyses

The W303 yeast strain was transformed with the above described plasmids or with the empty plasmids as negative controls. Cells were grown with shaking at 30 °C in SD-LEU medium for cultures transformed with the p415 plasmid and in SD-TRP medium for cultures with the p424 plasmid, and they were harvested by centrifugation at an OD_600_ of 1–2. Proteins were extracted from the cell pellets by alkaline hydrolysis as previously described [69]. The extracted proteins were separated on a 10% SDS-Laemmli-polyacrylamide gel, electrophoretically transferred to nitrocellulose membranes (Amersham Protran, GE Healthcare Life Science, Chicago, IL, USA), probed with anti-HA antibodies (Covalab, Bron, France) and the signals were revealed with a Clarity Western ECL Substrate kit (Bio-Rad, Hercules, CA, USA) using a Bio-Rad ChemiDoc XRS+ and Image Lab 5.2 software.

## 3. Results

### 3.1. Bioinformatic Analyses

#### 3.1.1. Identification of *L. infantum* DEAD-Box Proteins

We were primarily interested in *L. infantum* because it is the cause of visceral leishmaniasis, which is the most severe form of the disease, and consequently the proteins were of interest as potential targets for therapeutic drugs. Out of 102 sequences listed as helicases on the TriTryp website, we found 29 genes that contained the conserved motifs that were characteristic of DEAD-box proteins in *L. infantum*. We compared these sequences with those of other *Leishmania* species and discovered that LINF_080005700 was much longer in most other species of *Leishmania* and other trypanosomatids than for *L. infantum*. We inspected the upstream DNA sequence and found another ATG start codon in the same open reading frame that would yield a protein product of similar size to those of the other species. It contained an amino-terminus that was 181 amino acids longer than that listed on the TriTryp database for *L. infantum*, and it was possible that both long and short forms of the protein were expressed. Moreover, the *T. brucei* equivalent, Tb927.5.3600, was predicted to be expressed as both a long and short form [70]. Consequently, we used both forms for all subsequent analyses.

We compared our results with the previous *in silico* analyses of RNA helicases in trypanosomatids [71]. *L. major* and *L. infantum* are closely related, and the majority of the identified DEAD-box proteins were the same [72]. LmjF.05.0140 did not originally appear to exist in *L. infantum* but very recent work shows that the *L. infantum* homolog (LINF_050006300) was misclassified as a rRNA on the TriTrypDB database [73]. Consequently, it was not included in these alignments, but it encodes a DEAD-box protein that functions as a putative nucleolar RNA helicase II [74]. We found three more DEAD-box proteins in both organisms: LINF_200013600 (LmjF.20.0870), LINF_110006800 (LmjF.11.0190), and LINF_160005500 (LmjF.16.0050). Finally, the *L. infantum* equivalent of LmjF.10.0140, LINF_100006300, lacked the motifs characteristic of DEAD-box proteins. Thus, we identified 30 different DEAD-box genes in *L. infantum*, although the eIF4A-like *LINF_010012800* and *LINF_010012900* encoded the same protein. Thus, there were 29 different DEAD-box proteins, which includes LINF_050006300.

#### 3.1.2. Phylogenetic Relationship of the *L. infantum* Proteins and the Ded1/DDX3 Subfamily

**Figure 1:**
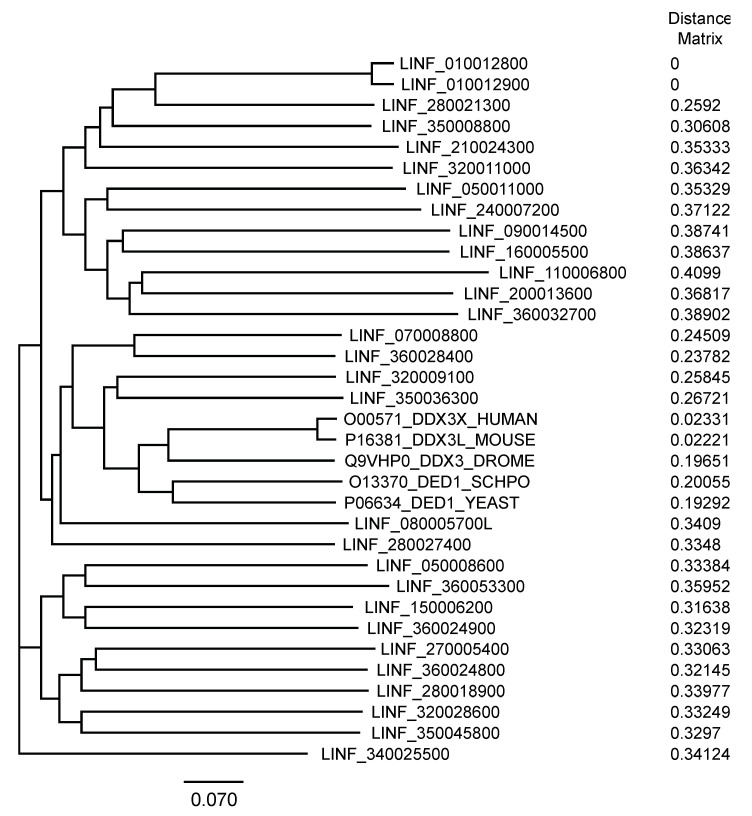
Phylogenetic tree of LINF and Ded1/DDX3 proteins. A neighbor-joining tree is shown with the branch lengths and distances as shown. The UniProt identifying numbers are shown for the Ded1 and DDX3 proteins, and the TriTryp numbers for *L. infantum*. The DDX3 proteins were from humans, mice and *Drosophila melanogaster*, and the Ded1 proteins from *Schizosaccharomyces pombe* and *S. cerevisiae*.

We used T-coffee to align these sequences against yeast Ded1 and four DDX3-like proteins that are the functional homologs—orthologs—of Ded1; which is, the four proteins are able to complement a yeast strain deleted for the essential *DED1* gene ([34] and reference therein). The phylogenetic tree result is shown in Figure 1. These data showed that the *Leishmania* proteins were highly divergent from the known Ded1/DDX3 proteins, but that the previously identified LINF_320009100 and to a lesser extent LINF_350036300 were the most closely related [75,76]. However, DEAD-box proteins are characterized by highly conserved RecA-like cores and highly variable flanking sequences [22]. It was unclear whether the former, the latter or both contained the distinguishing features of the Ded1/DDX3 subfamily.

Consequently, we repeated the T-coffee alignment with the core sequences alone starting from the highly conserved, but isolated, amino-terminal aromatic residue to the carboxyl-terminal end of motif VI. As expected, the phylogenetic tree was similar, but it showed less divergence (Supplementary Materials Figure S1A). The Ded1/DDX3 subfamily of proteins are characterized by amino-terminal, leucine-rich, nuclear-export signal (NES), an eIF4E binding motif, and a conserved GINF sequence, and they contain a conserved carboxyl-terminal RDYR sequence [77,78]. The flanking sequences of the LINF DEAD-box proteins varied enormously in length; nevertheless, we repeated the T-coffee alignments with the flanking sequences alone (Supplementary Materials Figure S1B). The results showed that LINF_320009100 again was the most closely related to the Ded1/DDX3 subfamily. The LINF_350036300 protein was more distantly related after LINF_070008800. Thus, both the RecA-like cores and flanking sequences provided distinguishing features, but the flanking sequences were more pronounced, probably in part because of their more variable sizes.

#### 3.1.3. Sequence Alignments of the DEAD-Box Proteins

**Figure 2:**
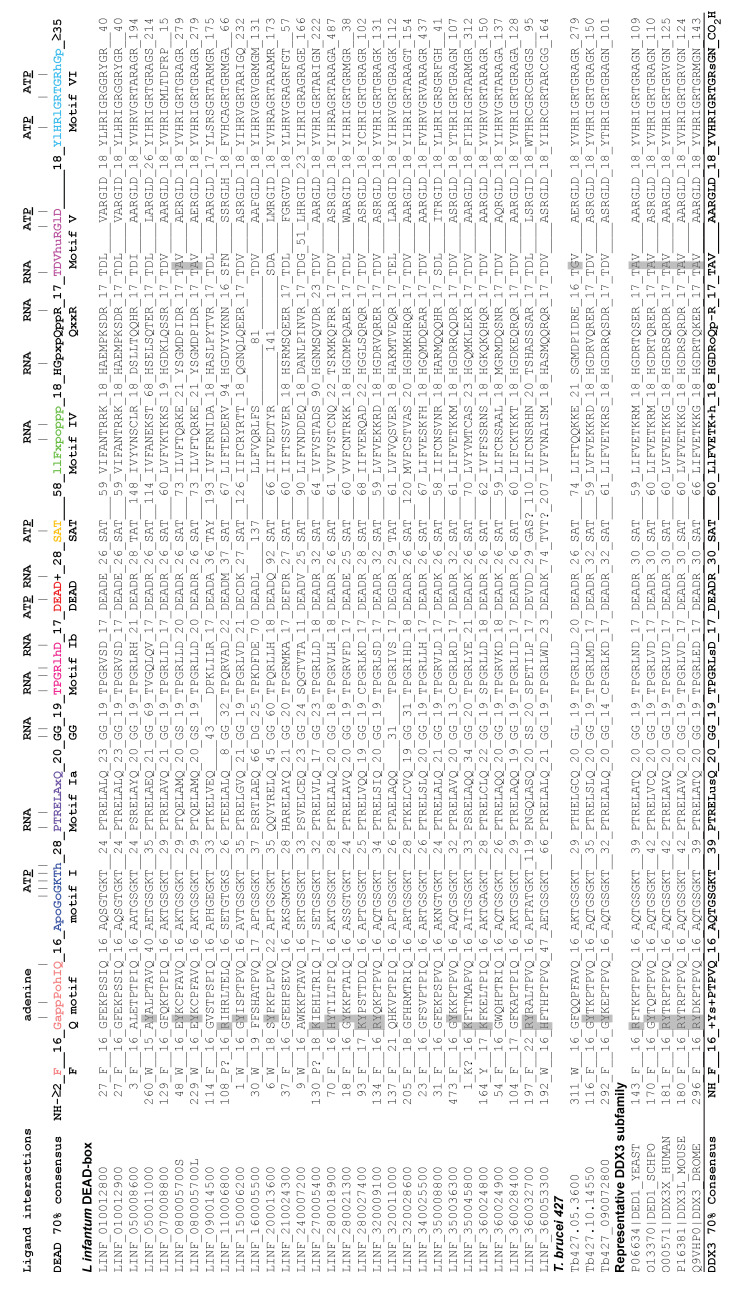
Alignment of the helicase cores of DEAD-box proteins of *Leishmania*. The protein sequence of the 28 different proteins is shown with representative members of the Ded1/DDX3 subfamily of proteins. Note that *LINF_010012800* and *LINF_010012900* encode the same protein, and both the long and short forms of *LINF_080005700* are shown. LINF_050006300 was found after this work was completed. The DEAD-box consensus was derived from 700 sequences from different organisms as previously described [56]. The capital case letters are conserved amino acids and the lowercase letters indicate functional conservation, where a is an aromatic group, l is an aliphatic residue, h is hydrophobic, o is an alcohol, + is positively charged, p is polar, u is tiny, and x is any residue. The numbers signify the typical distances between motifs. The interactions with the RNA and ATP are as shown, based on the solved crystal structures of the ligand-bound DEAD-box protein Vasa (PDB# 2DB3; [80]). However, these interactions are largely conserved in all the solved crystal structures of DEAD-box proteins. The underlined P shows interactions with the phosphates of ATP, while the adenine is from ATP. The helicase core is defined by 2–3 residues that extend from the amino terminus of the conserved phenylalanine of the Q motif and about 33–35 residues that extend from the carboxyl terminus of motif VI. Similarly, the Ded1/DDX3 subfamily consensus was derived from 96 of the original 229 unique sequences with the closest homology to yeast Ded1. Residues distinctive for this subfamily are shown in gray.

We aligned the RecA-like core sequences according to the parameters that we previously determined [56]. The conserved motifs generally appear at fixed distances from one another due to the steric constraints of the RecA-like core. This was used to refine alignments with divergent motifs. The results are shown in Figure 2. Most of the proteins had conserved motifs that conformed well to the consensus sequence that was previously determined for DEAD-box proteins in general [56]. These motifs form highly conserved interactions with the ligands, as determined from the solved crystal structures of a number of different DEAD-box proteins; these interactions are shown in Figure 2 ([56,79] and references therein). However, a number of proteins had poorly defined or missing motifs, which was most pronounced for LINF_160005500 and LINF_200013600. The QxxR motif appeared to be particularly divergent in the *Leishmania* proteins. However, it was possible that some of the differences were due to sequencing errors or strain variability. This may explain the 51 amino-acid insertion in motif V of LINF_240007200, but we very occasionally see an insertion at this position in other DEAD-box proteins as well. This region of motif V may be particularly vulnerable to insertions as the insert occurred between the regions that interacted with the RNA and ATP. We otherwise noted that the distances between motifs V, VI, and QxxR were conserved in this protein.

#### 3.1.4. Identification of Ded1/DDX3-Specific Characteristics

To facilitate comparisons, we determined the consensus sequence of 229 Ded1/DDX3 proteins that were identified from different organisms on the UniProtKB web site (https://www.uniprot.org/) using yeast Ded1 as bait and aligned using CLUSTAL W [81,82]. We further refined these alignments by using only those sequences with an E-value of 10^−113^ or less to yield 96 unique sequences that largely had the characteristics (defined above) of the Ded1/DDX3 subfamily. We used an arbitrary 70% consensus as a reference to further compensate for misidentified or ambiguous protein sequences (Figure 2). Consequently, the consensus sequences shown do not take into account the full diversity of sequences of potential members of this subfamily.

The core motifs of the Ded1/DDX3 subfamily were similar to those of other DEAD-box proteins in general, but there were significant differences. The Q motif consists of a loop-helix-loop-helix structure on the amino-terminus of the RecA-like core [83]. It contains an isolated aromatic group, which is often a phenylalanine, that is generally 17 residues upstream of the Q motif sequence GappPohIQ, where the glutamine is absolutely conserved, a is an aromatic group that is often a phenylalanine, p is a polar residue, o is an alcohol, and h is hydrophobic. The isolated phenylalanine forms stacking interactions with the proline, and the loop-helix-loop-helix forms a “cap” on RecA-like domain 1 [83]. The conserved glutamine interacts with the N6 and N7 positions of the adenine residue of ATP, and it is the only residue that can do so in the context of the DEAD-box protein structure. A glycine or small hydrophobic residue before the aromatic group is present in over 75% of the Q motifs of the DEAD-box sequences. The glycine occurs at a helix-loop transition, which is probably facilitated by a small residue. In contrast, we found that the Ded1/DDX3 subfamily had a positively charged residue, generally arginine, in over 85% of the sequences. This was true for a number of the LINF proteins as well, but the LINF_320009100 protein was the most similar.

Likewise, motif V is characterized by an aspartic acid in the second position in over 75% of the sequences and by a polar group in over 90%. This aspartic acid interacts with the first arginine of motif VI, which also makes highly conserved cation–π interactions with the conserved phenylalanine of motif IV that are important for the cooperative binding of RNA and ATP [84]. The Ded1/DDX3 subfamily had an alanine or small hydrophobic residue in over 75% of the sequences that would be expected to eliminate this interaction between motifs V and VI. Among the LINF proteins, only LINF_080005700 shared this characteristic. The Ded1/DDX3 subfamily also is thought to be distinguished by an insert between motif I and Ia that was proposed to affect RNA binding [31,85]. However, this region was highly variable in the LINF proteins and Ded1/DDX3 alignments, and regardless the insert is far from the RNA binding site in the solved crystal structures. It is not well conserved in plants and invertebrates [31]. Therefore, it did not appear to be a distinguishing feature. Likewise, other differences of the core sequences did not appear to be distinctive for the Ded1/DDX3 subfamily.

#### 3.1.5. Identification of Ded1/DDX3-Specific Motifs outside the RecA-Like Core

**Figure 3:**
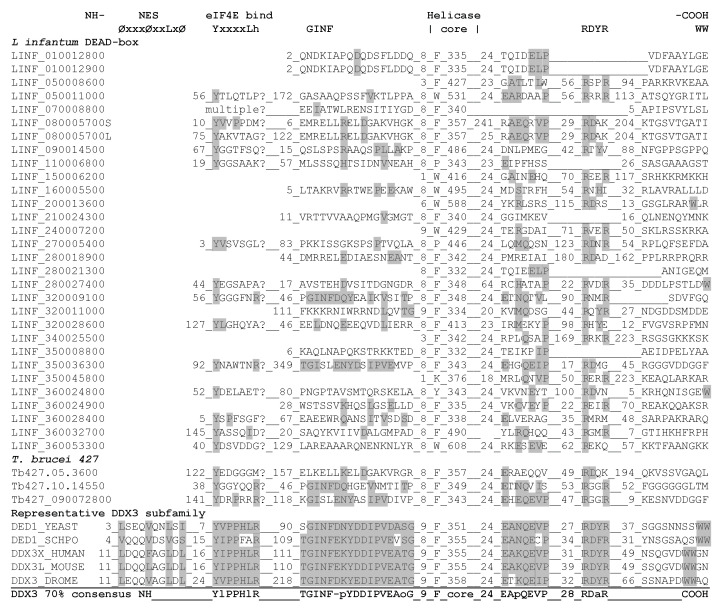
Alignment of the helicase flanking sequences of DEAD-box proteins of *Leishmania*. The consensus for the Ded1/DDX3 subfamily is as described in Figure 2, where it signifies a negatively charged residue. Aliphatic groups (I, L, V) are largely interchangeable, and we considered them equivalent in this figure. Ø denotes amino acids M, V, I, L, F, or W. The conserved sequences listed as characteristic of this subfamily (top) was based on previous work [77,78]. Distinctive residues for this subfamily are shown in gray. Note that the DDX3 70% consensus was based on a subset of the original sequences as indicated in Figure 2; hence it is not representative of the full diversity of annotated Ded1/DDX3 sequences obtained in Blasts.

The Ded1/DDX3 subfamily of proteins are cap-associated factors involved in translation initiation that actively shuttle between the nucleus and cytoplasm using the Mex67/TAP and XpoI/CrmI nuclear pore complexes (reviewed by [34,35]). The XpoI/CrmI-dependent export involves a leucine-rich NES near the amino-terminus with the sequence ØxxxØxxLxØ, where Ø denotes amino acids M, V, I, L, F, or W [86]. This region was clearly visible in the alignments of the five verified Ded1/DDX3 proteins (where V and L are interchangeable), but it was not found in the T-coffee alignments of the Ded1/DDX3 subfamily in general, probably because it lacked sufficient sequence conservation (Figure 3). Indeed, the NetNES 1.1 server was only able to predict the metazoan amino-terminal NES and not the yeast sequences [60]. The trypanosomatids contain the Ran-GTPase associated with the CrmI nuclear pore, but the sequence elements recognized are still poorly characterized [87]. Hence, it was unclear whether the leucine-rich NES was present in any of the LINF sequences.

Proteins involved in translation initiation often have one or more eIF4E binding motif; in the case of the Ded1/DDX3 subfamily, this motif is close to the amino terminus [78,88,89]. The motif is defined by the sequence YxxxxLh, where x is any amino acid [88]. The Ded1/DDX3 subfamily differed from this motif by having an arginine or positively charged residue as the terminal residue in over 80% of the sequences instead of a hydrophobic group (Figure 3). *Leishmania* are known to have often cryptic eIF4E binding motifs that are difficult to identify [90]. Nevertheless, we could find partial potential eIF4E binding motifs on the amino terminus for most of the LINF proteins, but LINF_080005700S, the short version, showed the best fit at a relevant distance from the amino terminus (Figure 3). Interestingly, LINF_070008800 showed multiple repeats of potential eIF4E binding motifs. However, it should be noted that trypanosomatids have at least six variant eIF4E proteins that are structurally different from those of other organisms due to the unusual cap-4 structure on their mRNAs (reviewed by [49,91,92]). Indeed, none of the tested *Leishmania* eIF4E proteins complement a yeast strain deleted for the essential eIF4E gene [93].

Shih et al. and Floor et al. found an amino-terminal motif consisting of the GINF sequence and a carboxyl-terminal motif consisting of RDYR that is characteristic of the Ded1/DDX3 subfamily through an alignment of six proteins [77,78]. We were able to expand on these results with our more extensive collection of sequences and compare them with our previous DEAD-box alignments [56]. The results are shown in Figure 3. The conserved residues of the GINF motif were characteristic of the Ded1/DDX3 subfamily, and they were more extensive than previously noted. The motif occurred at a fixed distance from the isolate aromatic group of the Q motif, which made its identification largely unambiguous. It is predicted to form a short α-helix [77]. None of the LINF sequences showed a strong conservation in this sequence, but LINF_320009100 and LINF_350036300 were the most similar.

The RDYR motif appeared at a more variable distance from motif VI, and it is thought to be important for the oligomerization of the protein [77]. Most of the LINF proteins had potential variants of this sequence as did the DEAD-box proteins in general, and consequently it did not appear to be a distinguishing feature of this subfamily (Figure 3). Moreover, it has been noted that a pair of tryptophans were often present at the carboxyl-terminus of the Ded1/DDX3 subfamily [77,78]. We found a tryptophan doublet within the last five residues of the carboxyl-terminus in about 70% of our aligned Ded1/DDX3 proteins and less often a tryptophan-aspartic acid (20%). The role(s) of these residues is unknown. Tryptophans were often present in our LINF alignments, but they were scattered at different positions and rarely appeared as doublets. Finally, we noted an additional motif with the sequence EApQEVP that appeared at a conserved distance after motif VI that existed in the Ded1/DDX3 subfamily but not in DEAD-box proteins in general (Figure 3). This sequence was most evident in LINF_080005700, LINF_320009100, and LINF_350036300.

From these data, and based on its similar size, we concluded that LINF_320009100 was the most likely ortholog of the Ded1/DDX3 subfamily. LINF_350036300 appeared to belong to the related DDX4 subfamily of proteins that includes *Drosophila* Vasa. Vasa has very occasionally shown partial complementation of a yeast strain deleted for the *DED1* gene, but only after very long incubation times, which also reflects the remarkable adaptability of yeast [94]. LINF_080005700 appeared to be a potential paralog of the Ded1/DDX3 subfamily because of its unusual motif V and Q motif, and because of the potential eIF4E binding and EApQEVP motifs. LINF_070008800 and LINF_360028400 lacked most of the distinguishing features of the Ded1/DDX3 subfamily despite their proximity in the phylogenetic tree.

#### 3.1.6. Comparisons between the *L. infantum* and *T. brucei* Ded1/DDX3-Like Proteins

To validate the selection of these proteins, we analyzed the equivalent genes from *T. brucei*. The Tb427.05.3600 protein was similar to LINF_080005700 in that it contained an unusual glycine in the second position of motif V (not a D or polar group; Figure 2), but it lacked the other characteristic features. In contrast, motif Ia in both proteins contained either a glutamine or a histidine in place of the highly conserved arginine in the third position; this residue makes conserved interactions with the RNA substrate, and it is an arginine in over 95% of the aligned DEAD-box and Ded1/DDX3-subfamily proteins (Figure 2). Both the glutamine and histidine substitutions would be expected to lose or significantly reduce the interactions with the RNA at this position. Indeed, a similar R276K mutation in human DDX3X is associated with medulloblastoma cancer [95]. Finally, both Tb427.10.14550 and Tb427_090072800 had partial conservation of the GINF and EApQEVP motifs, which was consistent with them being homologs of LINF_320009100 and LINF_350036300, respectively (Figure 3).

The nuclear localization signal (NLS) is poorly defined in the Ded1/DDX3 subfamily, but nuclear proteins of trypanosomatids are often characterized by a KRxR motif [96]. This motif was present in Tb427.05.3600 and LINF_080005700, but was not apparent in the other proteins. Moreover, Tb427.05.3600 has a predicted NES as well [96]. Nevertheless, Tb427.05.3600, Tb427.10.14550, and Tb427_090072800 are all enriched in the nuclear fractions of the tsetse fly midgut-form of *T. brucei* extracts [96]. These results were consistent with the identified proteins shuttling between the cytoplasm and nucleus and re-enforced their identification as potential Ded1/DDX3 homologs.

To facilitate subsequent analyses and comparisons, LINF_080005700 will be henceforth named LINF08L/S (long/short), LINF_320009100 is LINF32, LINF_350036300 is LINF35, Tb427.05.3600 is TRYP08, Tb427.10.14550 is TRYP32, and Tb427_090072800 is TRYP35.

### 3.2. Yeast Complementation

**Figure 4:**
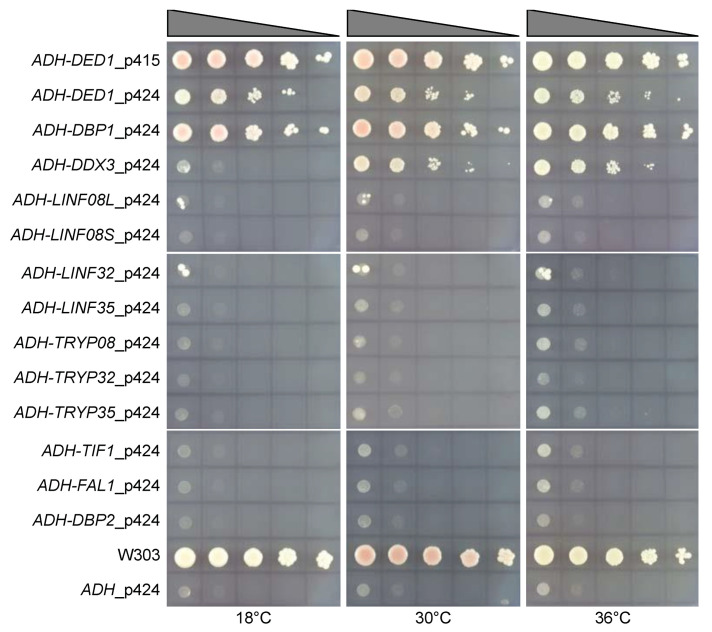
Complementation of the genes in a *ded1*-deletion strain. The listed genes in the HA-p415 centromeric or HA-p424 2µ plasmids were constitutively expressed in the *ded1::HIS* yeast strain from the strong *ADH* promoter, and then cultures were serially diluted by factors of 10 and spotted on SD plates containing 5-FOA that were subsequently incubated at the indicated temperatures. Plates were incubated three days at 30 °C and 36 °C, and for 7 days at 18 °C. W303 is a wildtype strain expressing endogenous *DED1*. *TIF1*, *FAL1*, and *DBP2* encode yeast DEAD-box proteins that are unrelated to the Ded1/DDX3 subfamily. DDX3X is the human protein and *DBP1* is the yeast paralog of *DED1* that suppresses the *DED1* deletion when overexpressed. *ADH*_p424 is the empty plasmid. Similar results were obtained with proteins expressed off the p415 plasmid.

The best evidence for a functional conservation of the Ded1/DDX3 subfamily is the ability to complement the deletion of the equivalent gene in yeast. Indeed, others and we have shown that proteins from organisms as diverse as *Drosophila*, mammals, and *S. pombe* are able to support growth in yeast strains deleted for the essential *DED1* gene ([34] and references therein). Therefore, we cloned the selected genes in yeast expression vectors with two, amino-terminal, HA tags under the control of the strong *ADH* promoter to test if the selected proteins were the orthologs of the Ded1/DDX3 subfamily [97]. The various constructs were then transformed into a yeast strain deleted for the chromosomal copy of the *DED1* gene (*ded1::HIS*) but that expressed Ded1 off a plasmid containing a *URA* marker [67]. Only cells that had lost the *URA* plasmid can grow when plated on agar medium containing 5-FOA, but only if the corresponding *TRP* or *LEU* plasmids expressed functional homologs of Ded1. As positive controls, we used the human *DDX3X* gene and a yeast paralog of *DED1*, *DBP1*, which partially compensates for the deletion of the *DED1* gene when overexpressed [98]. As negative controls, we used *TIF1* (eIF4A), *FAL1*, and *DBP2* genes, all of which encode unrelated yeast DEAD-box proteins. The results are shown in Figure 4.

Overexpression of the yeast Ded1 protein results in a dominant-negative growth phenotype [22,99]. This was apparent by the reduced growth of cultures expressing the *ADH-DED1*_p424, 2µ-multicopy, plasmid relative to the wildtype growth of W303 or the centromeric p415 plasmid (Figure 4). As expected, both human *DDX3X* and yeast *DBP1* partially compensated for the loss of the *DED1* gene, but the cells transformed with the *LINF* or *TRYP* genes showed no growth, although we did see a few isolated colonies. We recovered the plasmids from these isolated colonies and sequenced the genes; they all contained copies of *DED1*. This most likely resulted because the *URA* plasmid encoding *DED1* developed a mutation in the *URA* gene that inactivated its expression, which resulted in isolated cell growth on the 5-FOA plates. Therefore, none of the expressed *LINF* or *TRYP* genes complemented the *ded1* deletion. This was in contradiction with a previous publication that showed that the equivalent of *LINF32* and *LINF35* in *L. major*, LmjF.32.0400, and LmjF.35.3200, respectively, showed nearly wildtype growth [75]. However, these cultures were restreaked a second time on 5-FOA plates, and it was likely that the growth resulted from the above mentioned *DED1-URA* plasmids. Indeed, the expression of the tagged *L. major* LmjF32.0400 was not detected in yeast [75].

### 3.3. Protein Expression

**Figure 5:**
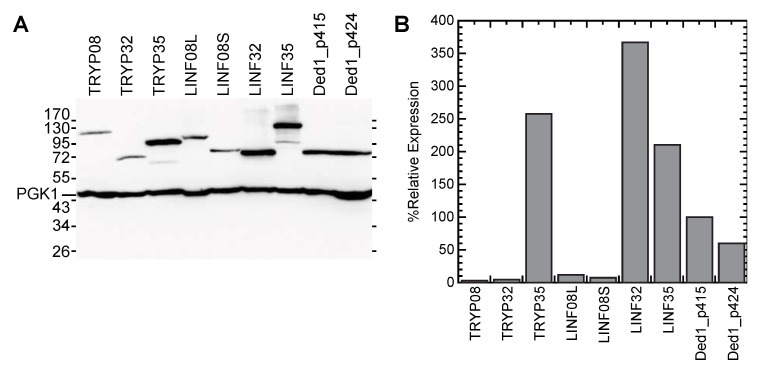
Expression of the proteins in yeast. The HA-tagged proteins in the p424 plasmid were expressed off the *ADH* promoter in the W303 yeast strain. (**A**) The proteins from the extracted cells were separated on a 10% SDS-PAGE, the separated proteins transferred to nitrocellulose membranes, and then visualized with IgG specific to the HA tag or PGK1. (**B**) The quantified values of the gels shown in (**A**). Variations in loading were adjusted relative to the PGK1, and then the values were normalized relative to the expression of HA-Ded1_p415.

A trivial explanation for the lack of complementation by the *LINF* or *TRYP* genes was that the proteins were poorly expressed or rapidly degraded. Therefore, we prepared cell cultures expressing the HA-tagged proteins in the wildtype W303 strain, extracted the proteins, separated them on SDS-PAGE, transferred the proteins to nitrocellulose membranes, and then probed with IgG specific for the HA tag. As a control, we probed the same membranes with IgG against the endogenously expressed PGK1 protein (YCR012W). The results are shown in Figure 5.

LINF32 and TRYP08 showed some variable expression levels between different preparations, but LINF32 was generally expressed in excess over Ded1 while TRYP08 was poorly expressed. The other proteins showed more consistent expression levels. Both LINF08S and LINF08L were very poorly expressed. The long form of LINF08, which is about 24% larger than the short form, seemed to be expressed slightly better. The much larger LINF35 was 2-fold more highly expressed than Ded1_p415 while the TRYP35 was 2.5-fold more expressed. Similar results were obtained when the proteins were expressed off the p415 or p424 plasmids. Thus, it was unlikely that the absence of complementation was due to the poor expression of LINF35 and TRYP35, but the other proteins were more ambiguous.

### 3.4. Synthetic Genes

Yeast genes tend to be rich in adenines and thymidines. Indeed, Ded1 has a G/C content of 46% (Table 1). In contrast, both *Trypanosoma* and *Leishmania* have much higher G/C content in their genes, and the proteins of interest had over 50% and 60%, respectively. This high G/C content could have interfered with the transcription or translation of the genes, although LINF35 was well expressed even with the highest G/C content of over 65% (Table 1). However, gene expression depends on the context of the G-C base pairs and on the presence of rare codons. Thus, it was possible that the variable or poor expression was caused both by the G/C content and by rare codons. Thus, we synthesized the *Leishmania* genes with a codon usage optimized based on *DED1*, and we then tested for their ability to complement the *ded1* deletion strain. We cloned the genes into plasmids with the strong *ADH* and the very strong *GPD* promoters; the latter has up to 10-fold higher expression levels than the *ADH* promoter [97]. The results are shown in Supplementary Materials Figure S2.

We obtained a few isolated colonies with *ADH-LINF08syn* and a more significant number of colonies with *GPD-LINF32syn*; we recovered the plasmids from these colonies and sequenced them as described above. In all cases we recovered the *DED1* gene. Hence, none of the *LINF* genes complemented the yeast strain deleted for the endogenous *DED1*. It remained possible that the proteins were not well expressed even off the strong *ADH* and very strong *GPD* promoters. Therefore, we prepared yeast extracts of the W303 strain transformed with genes as described above. The results are shown in Supplementary Materials Figure S3.

The synthetic *LINF* genes had very high expression levels of *LINF08Lsyn* and *LINF32syn* that was 3.5- and 5.4-fold higher, respectively, than for *DED1*. *LINF35syn* showed only 63% of the expression while *LINF08Ssyn* was expressed at about the same level as *DED1* (1.1-fold). Consequently, the *LINF* genes with the codons optimized for expression in yeast showed very different expression levels than the wildtype genes. Constructs under the control of the *GPD* promoter showed similar results. Nevertheless, none of the genes were able to complement the deletion of *DED1* even at these high levels of expression. The relative reduced expression of the *LINF35* synthetic gene was probably a result of increased protein degradation that resulted from very high expression [100].

### 3.5. Ded1 Chimeras

Our alignments indicated that both the catalytic cores and the amino- and carboxyl-terminal flanking sequences contained specific features characteristic of the Ded1/DDX3 subfamily. However, it seemed likely that the flanking sequences were further adapted for their cellular environment. For example, the amino-terminus of human DDX3X interacts with innate, immune, signaling factors that are not present in yeast Ded1 (reviewed by [101]). It was possible that the *LINF* and *TRYP* genes were similarly modified to optimize their functionality in the environment of the parasites. In contrast, we expected the catalytic cores to retain the same functionality as the enzymatic activities would be expected to be similar in all organisms. We previously showed that the catalytic cores of yeast DEAD-box proteins were only interchangeable between proteins with similar functionalities [22]. We reasoned that this might be the case for the trypanosomatids as well.

We based our constructions on sequence alignments, 3D modeling of the proteins and on our previous experience [22]. We used the catalytic cores of the wildtype *TRYP* genes and the optimized, synthetic, *LINF* genes, and we used the amino- and carboxyl-terminal sequences of Ded1. As a positive control, we made a chimera between human *DDX3X* and yeast *DED1*, as the DDX3X protein showed partial complementation in yeast (Figure 4). To push the system, we incubated the plates for over a week, and to facilitate comparisons we used the same series of cultures as shown in Figure 4. The results are shown in Supplementary Materials Figure S4.

At very long incubation times even the empty plasmid showed slight growth at 30 °C and 36 °C. This might reflect an adaptation by the yeast and by the weak expression of the endogenous *DBP1* gene [98]. The negative controls *TIF1*, *FAL1*, and *DBP2* similarly showed weak growth. The Ded1-DDX3 positive control showed slightly less growth then the intact DDX3X protein, which was probably the result of altered interactions between the core and flanking sequences. Except for the *DED1-TRYP32* and *DED1-TRYP35* chimeras, all of the *LINF* and *TRYP DED1* chimeras showed a very slight enhancement of growth that oddly was most apparent at 36 °C. The optimum growth temperature for yeast is around 30 °C [66]. Similarly, the wildtype *LINF32*, *LINF35*, *TRYP08*, and *TRYP35* constructs showed slightly better growth that was most apparent at 36 °C after long incubation times. Slight growth was previously observed with the DDX4 protein Vasa but at 30 °C [94]. Thus, the catalytic cores of the LINF and TRYP proteins seem to be optimized for higher temperatures than the human and yeast proteins. While the weak growth was indicative of a homology between the proteins, it was too weak to demonstrate that they were functional orthologs of the Ded1/DDX3 subfamily.

## 4. Discussion

Our sequence alignments indicate that LINF32 and TRYP32, also known as HEL67 [102], are the most likely orthologs of DDX3, LINF35 and TRYP35 the homologs of DDX4-like proteins, and LINF08 and TRYP08 are probably more distantly related paralogs of DDX3. The DDX4 proteins have only been characterized in metazoans, and they are involved in developmental regulation ([33] and references therein). They probably evolved after the branching of metazoans [103]; however, it remains possible that such proteins have emerged in trypanosomatids for similar reasons. This nevertheless remains conjuncture because none of the proteins complement a yeast strain deleted for *DED1*. Moreover, although the *Leishmania* genomes are highly conserved at the content and synteny levels, the encoded genes have a high divergence of functionality even between species (reviewed by [104]). Thus, it is possible that all the identified proteins are paralogs of DDX3 that have evolved other cellular functions. Alternatively, the different proteins may be optimized, and expressed, for the different cellular environments that exist within the insect or mammalian hosts, but they nevertheless retain the same functionality. Indeed, mammals encode at least two to three different DDX3 proteins that are not interchangeable (reviewed by [30,31,33]). Human DDX3Y is needed for normal spermatogenesis, and it is translated only in the testes; DDX3X is ubiquitously expressed. It is interesting to note that the plant *Nicotiana benthamiana* contains six DDX3-like homologs, which perhaps reflects the divergent types of cells (roots, leaves, flowers, etc.; [105]).

The Ded1/DDX3 subfamily is characterized by specific sequence motifs in the amino- and carboxyl-terminal domains involving the NES and eIF4E binding motif, and other motifs of undefined functionality. Only LINF32, LINF35, TRYP32, TRYP35, and to a lesser extent LINF08 partially share these characteristics. However, it should be emphasized that this is based on the consensus sequence of a selected subset of Ded1/DDX3 proteins that does not reflect the full diversity of sequences. In addition, the Ded1/DDX3 proteins have RecA-like cores with a noncanonical Q motif and motif V. Typically, the first amino acid of the Q motif is a glycine or other small residue, which is probably needed to facilitate the helix-loop transition in the structure. In the Ded1/DDX3 subfamily this residue is often an arginine followed by a tyrosine. Only LINF_360032700 and LINF32 share this characteristic. However, this position appears flexible as *S. pombe* has a more typical glycine as the first amino acid. Moreover, changing the arginine to glycine in yeast Ded1 has no apparent phenotype (our unpublished data). The aspartic acid to alanine change in the second position of motif V is more profound as it would be expected to eliminate an important interaction with the first arginine of motif VI and thereby introduce instability in the interactions between motifs, which may be important for the functionality of the Ded1/DDX3 proteins. Only LINF08 (an alanine) and TRYP08 (a glycine) shared this characteristic. However this change may not be critical as the alanine to arginine mutation in yeast yields only a slight growth phenotype (our unpublished data). However, we expect that the arginine to alanine change would alter the cooperative binding of ATP and RNA [84].

The *L. major* equivalents of LINF32 and LINF35 and LmjF32.0400 and LmjF35.3100, respectively, have a weak interaction with the eIF4E proteins LeishIF4E-1 and LeishIF4E-4 that is probably mediated by the mRNA in both the promastigotes and axenic amastigote-like cells, which is consistent with a role in translation [75,106]. However, in contrast to yeast Ded1, no direct contact was noted between the proteins and the *Leishmania* eIF4A, eIF4E, and eIF4G proteins [34,75]. LmjF35.3100 showed more variable interactions between the different eIF4E proteins and *Leishmania* forms that are consistent with it having a developmental role. However, pronounced defects in translation and cell growth were only noted when both of the equivalent *T. brucei* proteins were silenced in procyclic cells of *Trypanosoma* ([75] and reference therein). Consistent with this, the double knockout of *L. infantum LINF32* was viable although it was sensitive to heat stress and acidic pH, and consequently it was unable to undergo axenic amastigote differentiation [76,102]. LmjF35.3100 is more abundant in promastigotes while LmjF32.0400 is more prevalent in amastigotes [75]. LINF32 was likewise more prevalent in axenic amastigotes [107]. The equivalent proteins in *L. donovani*, LdBPK_080080 (LINF08), LdBPK_320410 (LINF32), and LdBPK_353150 (LINF35) were recovered in both promastigotes and amastigotes, but LdBPK_080080 showed a weak signal [108]. This might explain why only LINF32 and LINF35 were found in *L. infantum* promastigotes [109].

LINF32 also has been implicated in blocking antisense-dependent rRNA fragmentation in *L. infantum* and thereby reducing cell death under stress conditions [76]. Similarly, LINF32 plays a central role in mitochondrial proteostasis under conditions of stress [102]. Under these conditions, the *LINF32* knockout mutant has depolarized mitochondrial membranes that lead to mitochondrial fragmentation and cell death. Pull-down experiments reveal interactions with proteins involved in antioxidant and unfolded protein response, but also interactions with LINF35 [102]. The *L. donovani* null (knockout) mutations of LdBPK_320410 impair the infectivity and induce protective immunity against visceral leishmaniasis, and as with *L. infantum* they were unable to differentiate as axenic amastigotes [110]. Thus, the *Leishmania* DDX3-like proteins show significant functional divergence from their counterparts in other organisms.

LINF08 and TRYP08 remain an enigma, as there is little information in the literature. Both proteins appear to be derived from DDX3-like proteins, but they have amino acid substitutions in motif Ia that would be expected to reduce RNA binding. Consistent with this, the equivalent proteins of LINF08, LINF32, and LINF35 in *L. mexicana* (LmxM.08.0080, LmxM.31.0400, and LmxM.34.3100, respectively) are expressed in both amastigotes and promastigotes, but only LmxM.31.0400 and LmxM.34.3100 are recovered as proteins prominently crosslinked to mRNAs [111]. However, other LINF proteins have altered RNA-binding motifs as well, including LINF_090014500, LINF_110006800, LINF_200013600, LINF_240007200, LINF_320011000, LINF_320028600, and LINF_360032700. The LINF proteins LINF_090014500, LINF_110006800, LINF_160005500, and LINF_240007200 appear to be particularly divergent from the canonical DEAD-box consensus motifs. While this may partially reflect sequencing errors and strain variability, it also reflects the high degree of divergence of the LINF proteins from other eukaryotes. It remains to be seen whether they still function as ATP-dependent RNA binding proteins and RNA-dependent ATPases.

The functional roles of LINF08 and TRYP08 remain unclear, but RNA interference studies in *T. brucei* show that TRYP08 is of critical importance for the viability of the bloodstream form but not for the other forms [112]. Interestingly, the bloodstream loss-of-fitness group was strongly overrepresented by proteins associated with flagellar motility, which was not the case for the flagellated, procyclic, loss-of-fitness group [112]. Thus, TRYP08 plays a critical role at only specific phases of the life cycle. In contrast, TRYP35 is important throughout the lifecycle while TRYP32 plays a more minor role [112]. Double and triple knockdowns are required to determine if these three proteins have overlapping activities.

Further work is needed to characterize these proteins in order to determine their cellular role(s) and whether they are true homologs of the Ded1/DDX3 subfamily. This includes the enzymatic characterization of the purified proteins and deletions and mutations of the endogenous genes to reveal their functional importance at the different phases of the parasite. Nevertheless, it is clear that the *Leishmania* and *Trypanosoma* DEAD-box proteins are highly divergent from their eukaryote counterparts in other organisms, and thus they provide a rich source of potential, protein-specific, drug targets.

## Figures and Tables

**Table 1:** Properties of selected DDX3-like genes and expressed proteins ^a^.

Protein	Identification ^b^	MW (Da)	Size (aa)	%Identity ^c^	pKi ^d^	%G/C
Ded1_S.c.	P06634	65,550	605	100.0	8.22	46.1
Ded1_S.p.	O13370	69,756	636	68.0	8.80	45.8
DDX3X_Hu	O00571	73,241	662	62.0	7.17	45.9
DDX3L_Mu	P16381	73,138	660	61.0	7.17	50.7
DDX3_D.m.	Q9VHP0	85,078	798	61.0	7.70	56.4
LINF08S	LINF_080005700	75,345	686	31.0	6.70	61.2
LINF08L	LINF_080005700	93,670	867	31.0	6.14	61.4
LINF32	LINF_320009100	66,806	615	51.0	9.04	64.6
LINF35	LINF_350036300	100,533	925	46.0	6.40	65.4
TRYP08	Tb427.05.3600	103,320	949	33.0	6.20	50.2
TRYP32	Tb427.10.14550	66,562	619	51.0	9.25	55.2
TRYP35	Tb427_090072800	82,641	736	49.0	8.78	53.2

^a^ Data taken from DNAStrider 2.0. ^b^ Identification of UniProtKB or TriTrypDB. ^c^ Identity was based on 30 residues upstream of the isolated aromatic group of the Q motif, the entire RecA-like core, and 35 residues beyond motif VI. The flanking amino- and carboxyl-terminal sequences were not considered as their lengths were highly variable between proteins. ^d^ Log of the ionization constant.

## Data Availability

Not applicable.

## Supplementary Materials

The following are available online at https://www.mdpi.com/2073-4425/12/2/212/s1, Table S1: Oligonucleotides used in this study, Table S2: Constructs used in this study, Figure S1: Phylogenetic tree of LINF and Ded1/DDX3 proteins, Figure S2: Complementation of synthetic *LINF* genes optimized for expression in yeast, Figure S3: Expression of synthetic *LINF* genes optimized for yeast, Figure S4: Complementation of the yeast *ded1::HIS* strain.
