# Supplementary material for: The In Silico Identification of Potential Members of the Ded1/DDX3 Subfamily of DEAD-Box RNA Helicases from the Protozoan Parasite Leishmania infantum and Their Analyses in Yeast
Source: Genes (Basel). 2021 Feb 1;12(2):212. doi: 10.3390/genes12020212 (PMC7912733; doi:10.3390/genes12020212)
Supplement: Supplementary file 1 [file genes-12-00212-s001.pdf]

Supplementary Materials

The *in silico* identification of potential members of the Ded1/DDX3 subfamily of DEAD-box RNA helicases from the protozoan parasite *Leishmania infantum* and their analyses in yeast.

Molka Mokdadi, Yosser Zina Abdelkrim, Josette Banroques, Emmeline Huvelle, Rafeh Oualha, Hilal Yeter-Alat, Ikram Guizani, Mourad Barhoumi*, and N. Kyle Tanner*

Supplementary Table S1. Oligonucleotides used in this study

**Constructs**[a]	**Sequence (5'—3')**[b]
LINF08-1 (SpeI-NdeI–SalI/XhoI)	
LinJ.08_up1	GCCTAT**ACTAGTCATATG**CGTAAGCGGGCG
LinJ.08_low	GCCTAT**GTCGAC**GATCGTGGCACCAGTCACGGA
LINF08-2 (SpeI-NdeI–SalI/XhoI)	
LinJ08_up2	GCCTAT**ACTAGTCATATG**AACTACGCCTCCATTCGCA
LINF08-2syn (SpeI-NdeI–XhoI)	
LinJ08_up3	GCCTAT**ACTAGTCATATG**AATTATGCATCCATCAGAACCGA
LinJ08_low2	GCCTAT**CTCGAG**AATAGTAGCGCCAGT
LINF32 (SpeI-NdeI–XhoI)	
LinJ.32_up	GCCTAT**ACTAGTCATATG**TATAAGAATCAGGCGCAACCTC
LinJ.32_low	GCCTAT**CTCGAG**CTGACCAAAGACGTCAGATCGCA
LINF35 (SpeI-NdeI–XhoI)	
LinJ.35_up	GCCTAT**ACTAGTCATATG**GAATATATCAACGATAAGAAAATT TCCGA
LinJ.35_low	GCCTAT**CTCGAG**GAAGCCACCGTCGTCCACA
TRYP08 (XbaI-NdeI–SalI/XhoI)	
Tb427-08_up	GCCTAT**TCTAGACATATG**CCTAAACGTGAAAGGGA
Tb427-08_low	GCCTAT**GTCGAC**AAGCTGTGCCCCAACCGA
TRYP32 (SpeI-NdeI–XhoI)	
Tb427_32_up	GCCTAT**ACTAGTCATATG**CACGGCATGAATTTCGGCCA
Tb427_32_low	GCCTAT**CTCGAG**CATGGTGAGGCCTCCTCCTCCT
TRYP35 (SpeI-NdeI–SmaI)	
Tb427_35_up	GCCTAT**ACTAGTCATATG**AGTTTATCCCGCACAGA
Tb427_35_low	GCCTAT**CCCGGG**GAAGCCTCCGTCATCCACA
LINF08-2-GAT (SpeI-NdeI–XhoI)	
LinJ08_up3	GCCTAT**ACTAGTCATATG** AAT TATGCATCCATCAGAACCGA
LinJ08_low2	GCCTAT**CTCGAG**AATAGTAGCGCCAGT
LinJ08_GAT_up	AAGACTGGTTCCGGTgcAACTTTATGTTATGCTTTACCA
LinJ08_GAT_low	GCATAACATAAAGTTgcACCGGAACCAGTCTTAGCGGT
LINF08-1-GAT (SpeI-NdeI)	
LinJ08_up4	GCCTAT**ACTAGTCATATG**AGGAAAAGAGCCA
LINF32-GAT (SpeI-NdeI–XhoI)	
LinJ32_up2	GCCTAT**ACTAGTCATATG**TATAAAAATCAAGCCCA
LinJ32_low2	GCCTAT**CTCGAG**GAAACCGCCATCA
LinJ32_GAT_up	CAAACTGGTTCAGGTgcAACAGCTGCTTACTTGATACCT
LinJ32_GAT_low	AAGTAAGCAGCTGTTgcACCTGAACCAGTTTGAGCA
LINF35-GAT (SpeI-NdeI–XhoI)	
LinJ35_up2	GCCTAT**ACTAGTCATATG**GAATACATTAATGA
LinJ35_low2	GCCTAT**CTCGAG**GAAACCGCCATCA
LinJ35_GAT_low	AAGAATGCGGCAGTTgcACCAGAGCCTGTCTGA
LinJ35_GAT_up	CAGACAGGCTCTGGTgcAACTGCCGCATTCTTGA
**Chimera constructs**	
Ded1-5'-pUC18_fwd (SpeI-NdeI)	CGTTGTAAAACGACGGCCAGTGCCA**ACTAGTCATATG**GCTGAACTGAGCGAAC
Ded1-3'-pUC18_rev (XhoI)	GGAAACAGCTATGACCATGATTACG**CTCGAG**CCACCAAGAAGAGTTGTTTG
Ded1-5'-pUC18_fwd2 (XbaI-NdeI)	CGTTGTAAAACGACGGCCAGTGCCA**TCTAGACATATG**GCTGAACTGAGCGAAC
Ded1-3'-pUC18_rev2 (SalI)	GGAAACAGCTATGACCATGATTACG**GTCGAC**CCACCAAGAAGAGTTGTTTG
Ded1-DDX3-Ded1	
Ded1-5_rev-DDX3	TATGTGGAGGACAATCCTTACCAGAGGCGTC

Ded1-3_fwd-DDX3	AGCTAAACAAGAAGTCCCATCATTCTTGAAG
DDX3-core_fwd	CTCTGGTAAGGATTGTCCTCCACATATTGAAAG
DDX3-core_rev	AGAATGATGGGACTTCTTGTTTAGCTTCAACAAG
Ded1-LINF08-Ded1	
Ded1-5-Lj08_rev	TTGGTCTGGGAACATCCTTACCAGAGGCGTC
Ded1-3-Lj08_fwd	AGCGGAACAGAGAGTCCCATCATTCTTGAAG
Lj08-core_fwd	CTCTGGTAAGGATGTTCCCAGACCAATAAGATC
Lj08-core_rev	AGAATGATGGGACTCTCTGTTCCGCTCTCTC
Ded1-LINF32-Ded1	
Ded1-5-Lj32_rev	ATTCTGCTGGCTCAACATCCTTACCAGAGGCGTC
Ded1-3-Lj32_fwd	AACAAACCAAACCGTCCCATCATTCTTGAAG
Lj32-core_fwd	CTCTGGTAAGGATGTTGAGCCAGCAGAATCTTTTG
Lj32-core_rev	AGAATGATGGGACGGTTTGGTTTGTTTCTCTCAATAAAG
Ded1-LINF35-Ded1	
Ded1-5-Lj35_rev	CAACTGGTTTAACATCCTTACCAGAGGCGTC
Ded1-3-Lj35_fwd	ACACGGTCAAGAAGTCCCATCATTCTTGAAG
Lj35-core_fwd	CTCTGGTAAGGATGTTAAACCAGTTGAAGATTTTGC
Lj35-core_rev	AGAATGATGGGACTTCTTGACCGTGTTCACTAAAATAC
Ded1-TRYP08-Ded1	
Ded1-5-Tb08_rev	TCGGTCGTGGTGGATCCTTACCAGAGGCGTC
Ded1-3-Tb08_fwd	GGCAGAACAACAGGTCCCATCATTCTTGAAG
Tb08-core_fwd	CTCTGGTAAGGATCCACCACGACCGATGCGT
Tb08-core_rev	AGAATGATGGGACCTGTTGTTCTGCCCGCTC
Ded1-TRYP32-Ded1	
Ded1-5-Tb32_rev	ACACTGGTGCAATATCCTTACCAGAGGCGTC
Ded1-3-Tb32_fwd	TGAGACCAATCAGGTCCCATCATTCTTGAAG
Tb32-core_fwd	CTCTGGTAAGGATATTGCACCAGTGTTATCG
Tb32-core_rev	AGAATGATGGGACCTGATTGGTCTCATTGAG
Ded1-TRYP35-Ded1	
Ded1-5-Tb35_rev	CAACAGCATCAATATCCTTACCAGAGGCGTC
Ded1-3-Tb35_fwd	ACACGAACAGGAGGTCCCATCATTCTTGAAG
Tb35-core_fwd	CTCTGGTAAGGATATTGATGCTGTTGAAAGCTTTG
Tb35-core_rev	AGAATGATGGGACCTCCTGTTCGTGTTCACG

[a] Oligonucleotides used together are shown grouped

[b] Regions of hybridization are shown underlined, restriction sites are in bold and mutations are in lowercase.

Supplementary Table S2. Constructs used in this study

Name	Description	Source
B.S.	BlueScript (*AMP*)	
pUC18	(*AMP*)	
*ADH-2HA*_p415	*2HA*, *ADH/CYC1* (*LEU2/CEN*)	[1]
*ADH-2HA*_p424	*2HA*, *ADH/CYC1* (*TRP*/2μ)	[1]
*GPD-2HA*_p424	*2HA*, *GPD/CYC1* (*TRP*/2μ)	This study
*ADH-2HA-DED1*_p415	*2HA-DED1*, *ADH/CYC1* (*LEU2/CEN*)	[1]
*ADH-2HA-DED1*_p424	*2HA-DED1*, *ADH/CYC1* (*TRP*/2μ)	[1]
*GPD-2HA-DED1*_p424	*2HA-DED1*, *GPD/CYC1* (*TRP*/2μ)	This study
*ADH-2HA-DBP1*_p424	*2HA-DBP1*, *ADH/CYC1* (*TRP*/2μ)	[1]
*ADH-2HA-DDX3*_p424	*2HA-DDX3*, *ADH/CYC1* (*TRP*/2μ)	[2]
*ADH-2HA-TIF1*_p424	*2HA-TIF1*, *ADH/CYC1* (*TRP*/2μ)	[1]
*ADH-2HA-FAL1*_p424	*2HA-FAL1*, *ADH/CYC1* (*TRP*/2μ)	[1]
*ADH-2HA-DBP2*_p424	*2HA-DBP2*, *ADH/CYC1* (*TRP*/2μ)	[1]
*LINF08L*_B.S.	*LINF08L*, BlueScript (*AMP*)	This study
*LINF08S*_B.S.	*LINF08S*, BlueScript (*AMP*)	This study
*LINF32*_B.S.	*LINF32*, BlueScript (*AMP*)	This study
*LINF35*_B.S.	*LINF35*, BlueScript (*AMP*)	This study
*ADH-2HA-LINF08L*_p415	*2HA-LINF08L*, *ADH/CYC1* (*LEU2/CEN*)	This study
*ADH-2HA-LINF08S*_p415	*2HA-LINF08S*, *ADH/CYC1* (*LEU2/CEN*)	This study
*ADH-2HA-LINF32*_p415	*2HA-LINF32*, *ADH/CYC1* (*LEU2/CEN*)	This study
*ADH-2HA-LINF35*_p415	*2HA-LINF35*, *ADH/CYC1* (*LEU2/CEN*)	This study
*ADH-2HA-LINF08L*_p424	*2HA-LINF08L*, *ADH/CYC1* (*TRP*/2μ)	This study
*ADH-2HA-LINF08S*_p424	*2HA-LINF08S*, *ADH/CYC1* (*TRP*/2μ)	This study
*ADH-2HA-LINF32*_p424	*2HA-LINF32*, *ADH/CYC1* (*TRP*/2μ)	This study
*ADH-2HA-LINF35*_p424	*2HA-LINF35*, *ADH/CYC1* (*TRP*/2μ)	This study

*GPD-2HA-LINF08L*_p424	*2HA-LINF08L*, *GPD/CYC1* (*TRP*/2μ)	This study
*GPD-2HA-LINF08S*_p424	*2HA-LINF08S*, *GPD/CYC1* (*TRP*/2μ)	This study
*GPD-2HA-LINF32*_p424	*2HA-LINF32*, *GPD/CYC1* (TRP/2μ)	This study
*GPD-2HA-LINF35*_p424	*2HA-LINF35*, *GPD/CYC1* (*TRP*/2μ)	This study
*LINF08Lsyn*_B.S.	*LINF08Lsyn*, BlueScript (*AMP*)	This study
*LINF08Ssyn*_B.S.	*LINF08Ssyn*, BlueScript (*AMP*)	This study
*LINF32syn*_B.S.	*LINF32syn*, BlueScript (*AMP*)	This study
*LINF35syn*_B.S.	*LINF35syn*, BlueScript (*AMP*)	This study
*ADH-2HA-LINF08Lsyn*_p415	*2HA-LINF08Lsyn*, *ADH/CYC1* (*LEU2/CEN*)	This study
*ADH-2HA-LINF08Ssyn*_p415	*2HA-LINF08Ssyn*, *ADH/CYC1* (*LEU2/CEN*)	This study
*ADH-2HA-LINF32syn*_p415	*2HA-LINF32syn*, *ADH/CYC1* (*LEU2/CEN*)	This study
*ADH-2HA-LINF08Lsyn*_p424	*2HA-LINF08Lsyn*, *ADH/CYC1* (*TRP*/2μ)	This study
*ADH-2HA-LINF08Ssyn*_p424	*2HA-LINF08Ssyn*, *ADH/CYC1* (*TRP*/2μ)	This study
*ADH-2HA-LINF32syn*_p424	*2HA-LINF32syn*, *ADH/CYC1* (*TRP*/2μ)	This study
*ADH-2HA-LINF35syn*_p424	*2HA-LINF35syn*, *ADH/CYC1* (*TRP*/2μ)	This study
*GPD-2HA-LINF08Lsyn*_p424	*2HA-LINF08Lsyn*, *GPD/CYC1* (*TRP*/2μ)	This study
*GPD-2HA-LINF08Ssyn*_p424	*2HA-LINF08Ssyn*, *GPD/CYC1* (TRP/2μ)	This study
*GPD-2HA-LINF32syn*_p424	*2HA-LINF32syn*, *GPD/CYC1* (*TRP*/2μ)	This study
*GPD-2HA-LINF35syn*_ p424	*2HA-LINF35syn*, *GPD/CYC1* (*TRP*/2μ)	This study
*TRYP08*_B.S.	*TRYP08*, BlueScript (*AMP*)	This study
*TRYP32*_B.S.	*TRYP32*, BlueScript (*AMP*)	This study
*TRYP35*_B.S.	*TRYP35*, BlueScript (*AMP*)	This study
*ADH-2HA-TRYP08*_p424	*2HA-TRYP08*, *ADH/CYC1* (*TRP*/2μ)	This study
*ADH-2HA-TRYP32*_p424	*2HA-TRYP32*, *ADH/CYC1* (*TRP*/2μ)	This study
*ADH-2HA-TRYP35*_p424	*2HA-TRYP35*, *ADH/CYC1* (*TRP*/2μ)	This study
*GPD-2HA-TRYP08*_p424	*2HA-TRYP08*, *GPD/CYC1* (*TRP*/2μ)	This study
*GPD-2HA-TRYP32*_p424	*2HA-TRYP32*, *GPD/CYC1* (*TRP*/2μ)	This study
*GPD-2HA-TRYP35*_p424	*2HA-TRYP35, GPD/CYC1* (*TRP*/2μ)	This study
*DED1-LINF08Lsyn*_pUC18	*DED1-LINF08Lsyn-DED1*_pUC18	This study
*DED1-LINF08Ssyn*_pUC18	*DED1-LINF08Ssyn-Ded1*_pUC18	This study
*DED1-LINF32syn*_pUC18	*DED1-LINF32syn-DED1*_pUC18	This study
*DED1-LINF35syn*_pUC18	*DED1-LINF35sy-DED1*_pUC18	This study
*DED1-TRYP08*_pUC18	*DED1-TRYP08-DED1*_pUC18	This study
*DED1-TRYP32*_pUC18	*DED1-TRYP32-DED1*_pUC18	This study
*DED1-TRYP35*_pUC18	*DED1-TRYP35-DED1*_pUC18	This study
*ADH-2HA-DED1-DDX3*_p424	*ADH-2HA-DED1-DDX3-DED1*_p424	This study
*ADH-2HA-DED1-LINF08Lsyn*_p424	*ADH-2HA-DED1-LINF08Lsyn-DED1*_p424	This study
*ADH-2HA-DED1-LINF08Ssyn*_p424	*ADH-2HA-DED1-LINF08Ssyn-DED1*_p424	This study
*ADH-2HA-DED1-LINF32syn*_p424	*ADH-2HA-DED1-LINF32syn-DED1*_p424	This study
*ADH-2HA-DED1-LINF35syn*_p424	*ADH-2HA-DED1-LINF35syn-DED1*_p424	This study
*ADH-2HA-DED1-TRYP08*_p424	*ADH-2HA-DED1-TRYP08-DED1*_p424	This study
*ADH-2HA-DED1-TRYP32*_p424	*ADH-2HA-DED1-TRYP32-DED1*_p424	This study
*ADH-2HA-DED1-TRYP35*_p424	*ADH-2HA-DED1-TRYP35-DED1*_p424	This study
*GPD-2HA-DED1-DDX3*_p424	*GPD-2HA-DED1-DDX3-DED1*_p424	This study
*GPD-2HA-DED1-LINF08Lsyn*_p424	*GPD-2HA-DED1-LINF08Lsyn-DED1*_p424	This study
*GPD-2HA-DED1-LINF08Ssyn*_p424	*GPD-2HA-DED1-LINF08Ssyn-DED1*_p424	This study
*GPD-2HA-DED1-LINF32syn*_p424	*GPD-2HA-DED1-LINF32syn-DED1*_p424	This study
*GPD-2HA-DED1-LINF35syn*_p424	*GPD-2HA-DED1-LINF35syn-DED1*_p424	This study
*GPD-2HA-DED1-TRYP08*_p424	*GPD-2HA-DED1-TRYP08-DED1*_p424	This study
*GPD-2HA-DED1-TRYP32*_p424	*GPD-2HA-DED1-TRYP32-DED1*_p424	This study
*GPD-2HA-DED1-TRYP35*_p424	*GPD-2HA-DED1-TRYP35-DED1*_p424	This study

1. Tanner, N.K., Cordin, O., Banroques, J., Doere, M. and Linder, P. (2003) The Q motif: a newly identified motif in DEAD box helicases may regulate ATP binding and hydrolysis. *Mol Cell*, **11**, 127-138. 10.1016/s1097-2765(03)00006-6
2. Senissar, M., Le Saux, A., Belgareh-Touze, N., Adam, C., Banroques, J. and Tanner, N.K. (2014) The DEAD-box helicase Ded1 from yeast is an mRNP cap-associated protein that shuttles between the cytoplasm and nucleus. *Nucleic Acids Res*, **42**, 10005-10022. 10.1093/nar/gku584

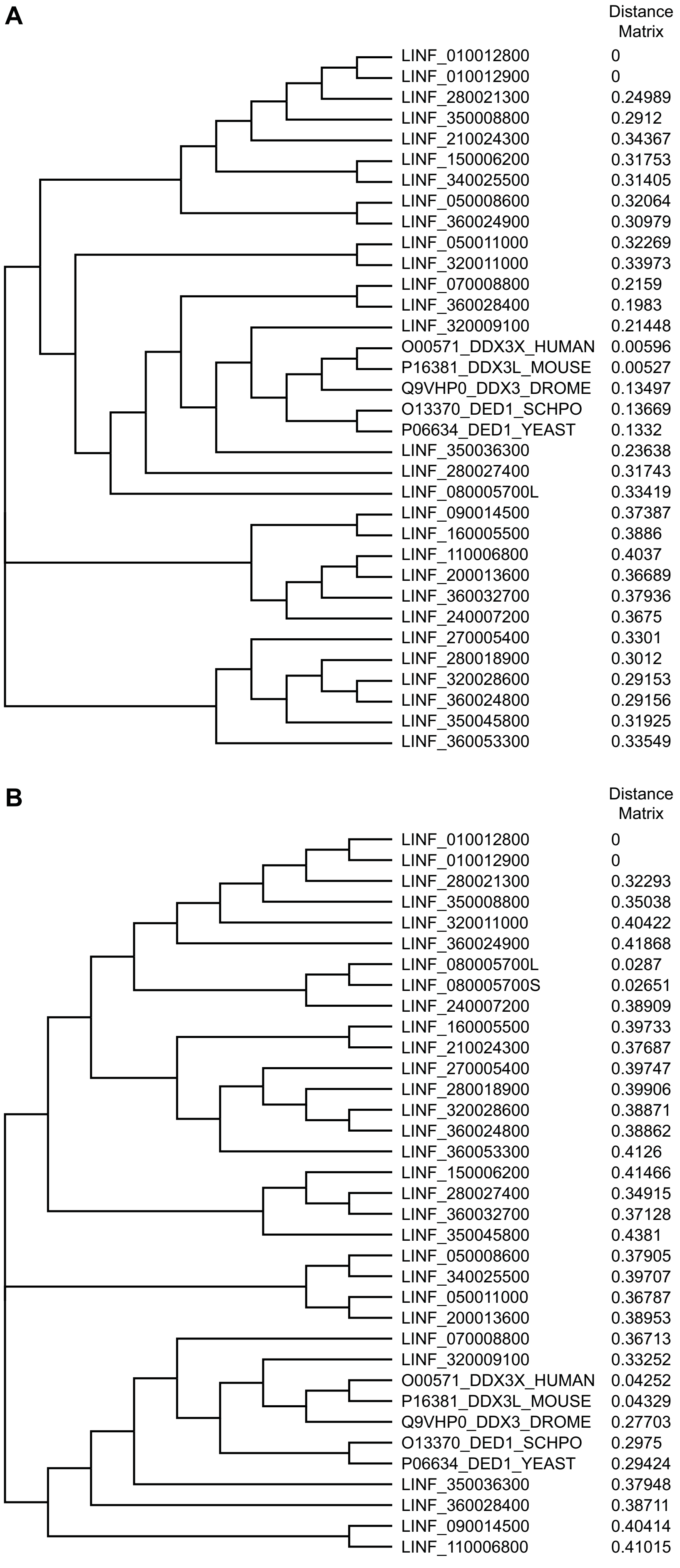
A
Distance Matrix
LINF_010012800 0
LINF_010012900 0
LINF_280021300 0.24989
LINF_350008800 0.2912
LINF_210024300 0.34367
LINF_150006200 0.31753
LINF_340025500 0.31405
LINF_050008600 0.32064
LINF_360024900 0.30979
LINF_050011000 0.32269
LINF_320011000 0.33973
LINF_070008800 0.2159
LINF_360028400 0.1983
LINF_320009100 0.21448
O00571_DDX3X_HUMAN 0.00596
P16381_DDX3L_MOUSE 0.00527
Q9VHP0_DDX3_DROME 0.13497
O13370_DED1_SCHPO 0.13669
P06634_DED1_YEAST 0.1332
LINF_350036300 0.23638
LINF_280027400 0.31743
LINF_080005700L 0.33419
LINF_090014500 0.37387
LINF_160005500 0.3886
LINF_110006800 0.4037
LINF_200013600 0.36689
LINF_360032700 0.37936
LINF_240007200 0.3675
LINF_270005400 0.3301
LINF_280018900 0.3012
LINF_320028600 0.29153
LINF_360024800 0.29156
LINF_350045800 0.31925
LINF_360053300 0.33549
B
Distance Matrix
LINF_010012800 0
LINF_010012900 0
LINF_280021300 0.32293
LINF_350008800 0.35038
LINF_320011000 0.40422
LINF_360024900 0.41868
LINF_080005700L 0.0287
LINF_080005700S 0.02651
LINF_240007200 0.38909
LINF_160005500 0.39733
LINF_210024300 0.37687
LINF_270005400 0.39747
LINF_280018900 0.39906
LINF_320028600 0.38871
LINF_360024800 0.38862
LINF_360053300 0.4126
LINF_150006200 0.41466
LINF_280027400 0.34915
LINF_360032700 0.37128
LINF_350045800 0.4381
LINF_050008600 0.37905
LINF_340025500 0.39707
LINF_050011000 0.36787
LINF_200013600 0.38953
LINF_070008800 0.36713
LINF_320009100 0.33252
O00571_DDX3X_HUMAN 0.04252
P16381_DDX3L_MOUSE 0.04329
Q9VHP0_DDX3_DROME 0.27703
O13370_DED1_SCHPO 0.2975
P06634_DED1_YEAST 0.29424
LINF_350036300 0.37948
LINF_360028400 0.38711
LINF_090014500 0.40414
LINF_110006800 0.41015

Supplementary Figure S1. Phylogenetic tree of LINF and Ded1/DDX3 proteins. A neighbor-joining tree is shown without distance corrections and with cladogram branch lengths to facilitate viewing. The distances are as shown. (**A**) Core sequences consisting of the amino-terminal, isolated, aromatic group to the end of motif VI. (**B**) Flanking sequences consisting of the fused amino- and carboxyl-terminal sequences and excluding the cores sequences used in **A**.

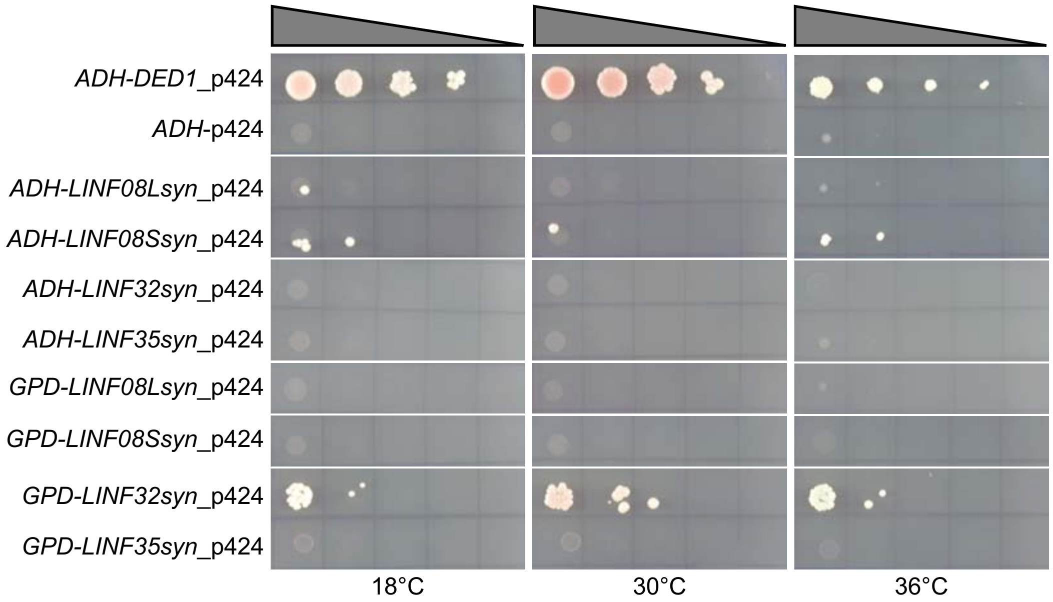

Supplementary Figure S2. Complementation of synthetic *LINF* genes optimized for expression in yeast. The yeast strain *ded1::HIS* was transformed with the indicated genes and grown in SD-TRP medium. Cultures were then serial diluted and spotted on SD plates containing 5-FOA. Plates were incubated for 6 days at 18°C and for 3 days at 30°C and 36°C. The isolated colonies that grew with the *LINF* genes contained the *DED1* gene, and hence no *LINF*-specific complementation was detected.

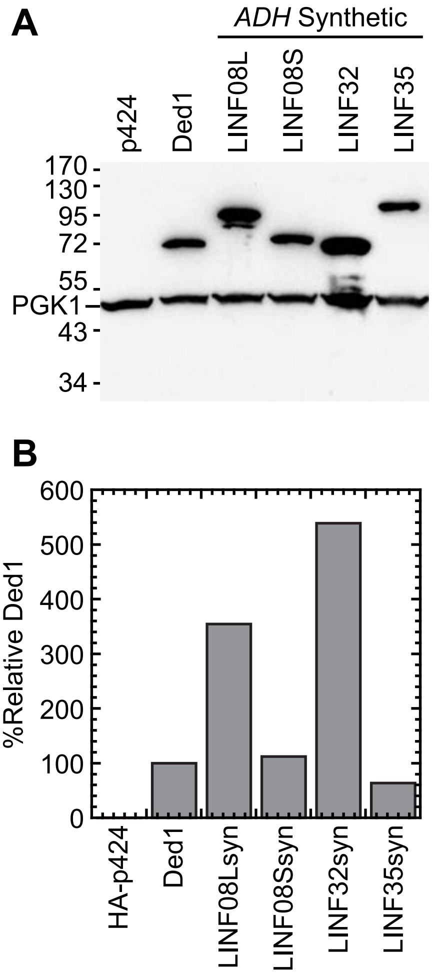

Supplementary Figure S3. Expression of synthetic *LINF* genes optimized for yeast. The HA-tagged proteins in the p424 plasmid were expressed off the *ADH* promoter in the W303 yeast strain. (**A**) The proteins from the extracted cells were separated on an 10% SDS-PAGE, the separated proteins transferred to nitrocellulose membranes and then visualized with IgG specific to the HA tag or PGK1. (**B**) The quantified values of the gels shown in (A). Variations in loading were adjusted relative to the PGK1, and then the values were normalized relative to the expression of HA-Ded1.

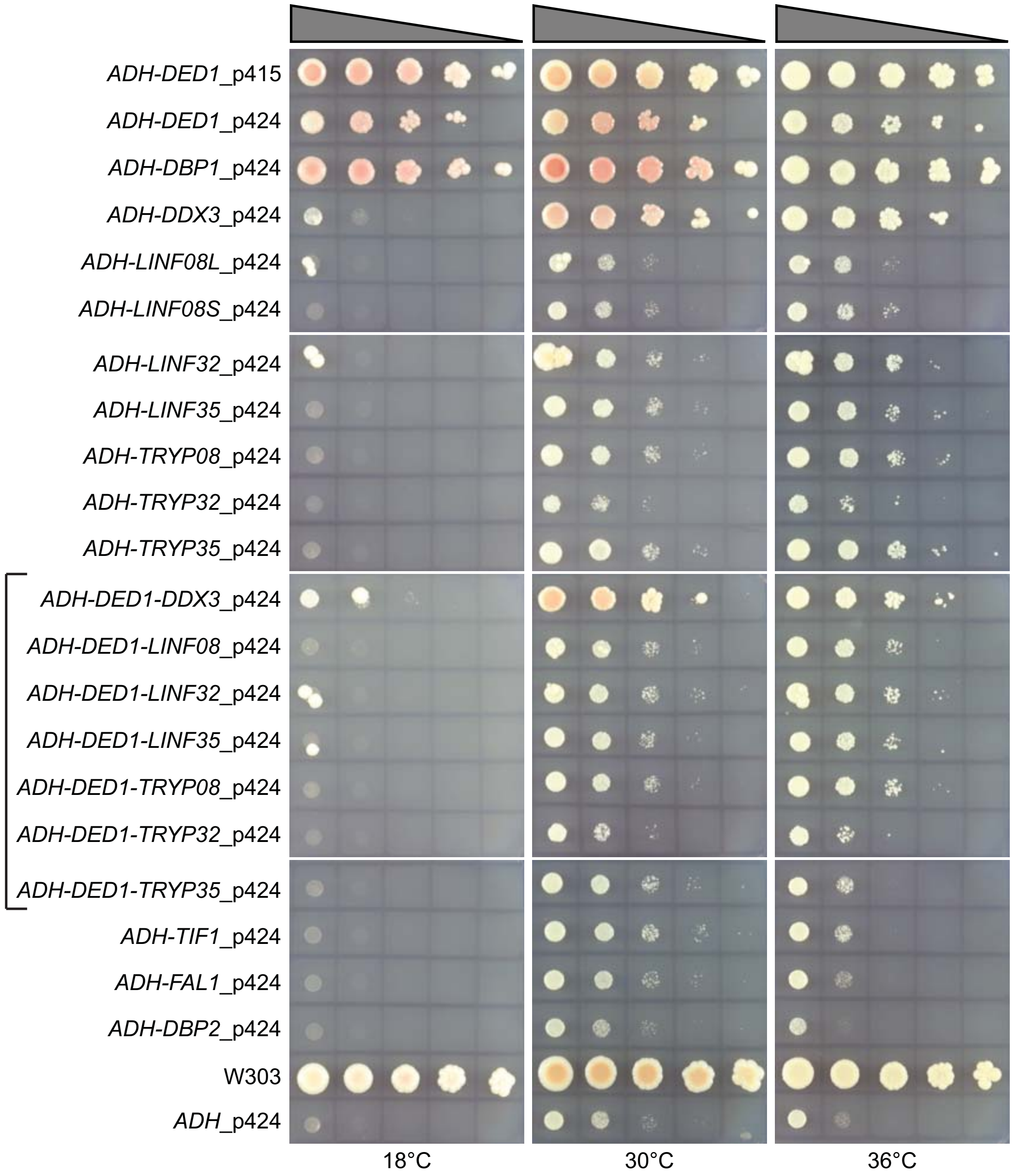

Supplementary Figure S4. Complementation of the yeast *ded1::HIS* strain. The chimeras with the LINF and TRYP catalytic cores and the Ded1 flanking sequences are shown bracketed. They contained the RecA-like catalytic cores of the indicated LINF and TRYP proteins and the amino- and carboxyl-terminal sequences of yeast Ded1. The plates are the same as those shown in Figure 4 except for the chimeras. Plates were incubated 7 days at 30°C and 36°C, and for 10 days at 18°C. The large isolated colonies in the *LINF* and *TRYP* lanes contained the *DED1* plasmid, which is most apparent at 18°C.
